# Epigenetic Modifications and Potential New Treatment Targets in Diabetic Retinopathy

**DOI:** 10.1155/2014/789120

**Published:** 2014-08-03

**Authors:** Lorena Perrone, Carmela Matrone, Lalit P. Singh

**Affiliations:** ^1^EA 7281 R2D2, Medical School, Auvergne University, 63000 Clermont-Ferrand, France; ^2^Department of Biomedicine, University of Aarhus, 8200 Aarhus, Denmark; ^3^Departments of Anatomy/Cell Biology and Ophthalmology, Wayne State University School of Medicine, Detroit, MI 48201, USA

## Abstract

Retinopathy is a debilitating vascular complication of diabetes. As with other diabetic complications, diabetic retinopathy (DR) is characterized by the metabolic memory, which has been observed both in DR patients and in DR animal models. Evidences have provided that after a period of poor glucose control insulin or diabetes drug treatment fails to prevent the development and progression of DR even when good glycemic control is reinstituted (glucose normalization), suggesting a metabolic memory phenomenon. Recent studies also underline the role of epigenetic chromatin modifications as mediators of the metabolic memory. Indeed, epigenetic changes may lead to stable modification of gene expression, participating in DR pathogenesis. Moreover, increasing evidences suggest that environmental factors such as chronic hyperglycemia are implicated DR progression and may also affect the epigenetic state. Here we review recent findings demonstrating the key role of epigenetics in the progression of DR. Further elucidation of epigenetic mechanisms, acting both at the cis- and trans-chromatin structural elements, will yield new insights into the pathogenesis of DR and will open the way for the discovery of novel therapeutic targets to prevent DR progression.

## 1. Introduction

Clinical data clearly demonstrate that early metabolic control is the most relevant factor to prevent the diabetic complications while prolonged hyperglycemia leads to long-lasting detrimental consequences that are not inhibited when the glycemic control occurs later [1, 2]. This phenomenon is defined as “metabolic memory.” Recent studies provided new insights suggesting that epigenetic alterations may be responsible, at least in part, for metabolic memory, also described as glycemic memory.

Epigenetic mechanisms influence gene expression and function without modification of the base sequence of DNA and may be reversible, heritable, and influenced by the environment [3]. Epigenetic modifications include DNA methylation, posttranslational histone modifications, chromatin remodeling, and deployment of noncoding RNA [3, 4]. Epigenetic alterations participate in pathologic responses such as inflammation and neurodegeneration, which contribute to the progression of diabetic retinopathy (DR) [5].

## 2. Prolonged Hyperglycemia and Metabolic Memory in DR

Clinical studies clearly demonstrate that an early glycemic control inhibits and may revert DR. The early treatment diabetic retinopathy study (ETDRS) demonstrated that visual loss is infrequent in early treated diabetic patients that received an appropriate glucose control [6]. Good glycemic control can block DR progression. However, the effects of good glycemic control on DR are not immediate. Several years of good glycemic control are necessary in order to inhibit DR progression. In addition, DR progression does not benefit of a good glycemic control after a profound period of poor glycemic control, suggesting a “metabolic memory” phenomenon. In addition, it is still an issue in the early prevention and treatment of vascular and microvascular complications in type-2 diabetes even in the presence of good glycemic control [7]. Indeed, recent studies show that early glucose control does not prevent the vascular and microvascular complications in diabetes [7]. On the other hand, recent studies are suggesting that advanced glycation end products (AGEs) formation has a major impact on DR progression compared to hyperglycemia [8]. Thus, it is important to develop animal models to unveil the molecular mechanisms implicated in the “metabolic” memory in RD. A dog model of diabetic retinopathy has been developed: diabetic dogs received poor diabetic control for 2,5 years followed by 2,5 years of good glycemic control [9]. In this animal model DR was absent after the 2,5 years of poor glycemic control, while DR appeared later despite the good glycemic control, demonstrating the concept of “metabolic memory” [9]. Several studies have been carried out to demonstrate in mice models the presence of metabolic memory. Streptozotocin-diabetic rats were maintained for 6 months in poor glycemic control followed by 6 months of good glycemic control [10]. The good glycemic control was not sufficient to block the microvascular dysfunction due to the previous poor glycemic control [10], further confirming that vascular alterations occur early in DR and maintain the metabolic memory.

In this review, we summarize the recent studies demonstrating the role of epigenetic alterations as mediators of the metabolic memory in DR progression. The studies have been carried out in the animal models described above, where the animals were maintained in poor glycemic control followed by a good glycemic control.

## 3. Vascular Dysfunction and Metabolic Memory in DR Progression

Vascular alterations play a key role in DR progression [11]. Indeed, clinical trial is demonstrating the role of retinal neovascularization caused by disequilibrium between pro- and antiangiogenic factors in DR progression [12]. The role of endothelial cells in promoting the metabolic memory in DR is still debated, since some studies underlined that endothelial cells show low proliferation rate [13, 14], suggesting that endothelial dysfunction may be recovered by blocking endothelial proliferation [12]. On the other hand, recent studies demonstrated that not only endothelial cells are implicated in vascular dysfunction in DR and that inflammation and retinal pericytes loss play a role in DR [15]. This study demonstrated that inflammatory mediators are early induced in DR, leading to pericytes dysfunction [15]. In addition, the duration of initial hyperglycemia as well as the duration of glycemic control after high glucose exposure is important for the modulation of inflammatory factors and subsequent pericytes alterations [15]. Indeed, only 8 days of normal glucose after 4 days of high glucose exposure showed amelioration in the production of proinflammatory factors [15]. Another study demonstrated that inflammation and subsequent vascular dysfunction are implicated in promoting the metabolic memory in DR by inducing epigenetic alterations [16]. Indeed, exposure to high glucose induces epigenetic alterations on cox2 promoter, leading to enhanced cox2 expression in retinal endothelial cells [16]. Thus, epigenetic alterations seem to occur early in the retinal microvascular system [16, 17], participating in the metabolic memory and promoting DR. In agreement, other epigenetic alterations occur early in the diabetic retina affecting the microvascular system and promoting the metabolic memory and DR progression [18]. Indeed, the miRNAs upregulating the expression of the vascular endothelial growth factor (VEGF) are early induced in DR, promoting neovascularization and confirming the role of epigenetic alterations in DR progression [18]. These studies are demonstrating that epigenetic alterations occur early in the microvascular system of the diabetic retina, promoting the metabolic memory and participating in DR progression [10, 16–18].

## 4. Epigenetics and Transcriptional Regulation

The chromatin structure regulates gene expression: transcriptional activity decreases with increased chromatin density while enhanced transcriptional activity is associated with loosening of chromatin structure [3]. Such changes in the state of chromatin are modulated by DNA methylation, histone modification, and noncoding RNAs.

**Figure 1:**
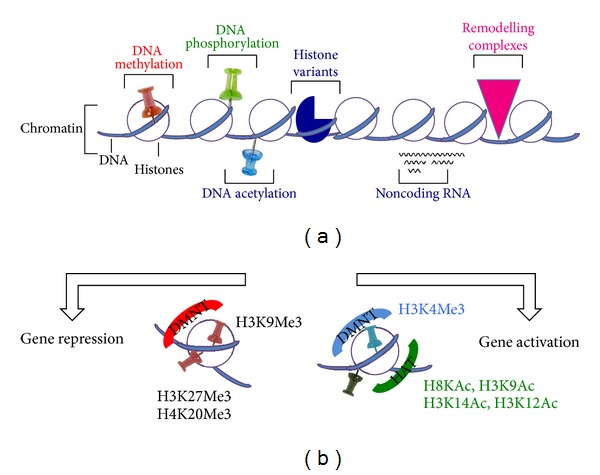
Scheme of histone and epigenetic modifications. (a) Chromatin structure can be modified in 5 different ways: DNA methylation, histone modification, remodeling by chromatin-remodeling complexes, insertion of histone variants, and the effects of noncoding RNAs (ncRNAs). (b) Histone modifications such as H3 lysine-9 methylation (H3K9me) or H3 lysine-27 methylation (H3K27me) result generally in gene repression, whereas H3 lysine-9/14 acetylation (H3KAc) and H3 lysine-4 methylation (H3K4me) are generally activation marks.

DNA methyltransferases (DNMTs) add a methyl group at the 5-position of cytosine converting the cytosine to 5-methylcytosine (5-^m^C) [3]. 5-^m^C is considered as the fifth base of the genome as well as 5-(hydroxymethyl) cytosine (5-h^m^C), the sixth base of the genome [19, 20]. In addition, 5-formyl-cytosine and 5-carboxyl-cytosine have also been recently characterized [21, 22]. The CpG dinucleotide is the most frequently targeted site of DNA methylation, which is important for chromatin condensation and gene transcription. In general, CpG methylation silences genes via chromatin closing while DNA demethylation results in enhanced transcriptional activity. Nonetheless, it has been recently described that the functional effects of DNA methylation are differentially modulated by the genomic context [23] (Figure 1(a)).

The principle DNA methyltransferases in mammals are DNMT1, DNMT3a, and DNMT3b [24]. DNMT1 is considered to be involved in copying and propagating methylation patterns to daughter cells during DNA replication at S phage and also involved in methylation maintenance (Figure 1(b)). On the other hand, DNMT3a and DNMT3b are needed for de novo DNA methylation of unmethylated CpG dinucleotides and required for the establishment of genomic methylation patterns during development. A mutation in any of these DNMTs is lethal suggesting the importance of maintaining DNA methylation in organismal survival. S-Adenosylmethionine (SAM) is the methyl donor used by methyltransferases in both DNA and histone methylation [25, 26]. It has been shown that circulating SAM level is reduced in patients with diabetic retinopathy [27], suggesting that alterations in DNA and/or histone methylation patterns may occur in DR.

Histones also can present various posttranslational modifications, including acetylation, methylation, phosphorylation, ubiquitination, and sumoylation. These modifications occur mostly within the N-terminal tails of histones (H2A, 2B, H3, and H4) not only protruding from the surface of the nucleosome, but also on its core region [28]. These modifications establish the histone code, which is an epigenetic mechanism involved in various physiologic and pathological phenomena. In general, histone acetylation, especially at H3 and H4 lysine residues, correlates with transcriptional activity, while histone deacetylation and methylation inhibit gene expression (Figure 1(b)).

Main histone acetyl transferases (HAT), which use acetyl-CoA as a substrate, are p300/CBP and pCAF, while histone deacetylases are HDACs and sirtuins [25]. Acetyl-CoA is generated in the mitochondrial tricarboxylic acid (TCA) pathway during glucose metabolism; therefore, diabetes and chronic hyperglycemia will have a profound influence on the bioavailability of this histone modification substrate and chromatin remodeling. Specifically, defined sites of acetylation of histone tails are considered to occur at transcriptionally active sites at gene promoters: histone H3 at lysine 9 (H3K9Ac), histone H3 at lysine 14 (H3K14Ac), histone H4 at lysine 8 (H4K8Ac), and histone H4 at lysine 12 (H4K12Ac). In addition, trimethylation of histone H3 at lysine 4 (H3K4Me3) by MLL (mixed lineage lymphoma) 5 methyltransferase also activates gene transcription by retinoic acid and under hyperglycemia via O-GlcNAcylation [29]. Conversely, histone methyltransferases that target H3K9Me3, H3K27Me3, and H4K20Me3 and inhibit transcription and their demethylases also play critical roles in gene expression and cell viability [30, 31] (Figure 1(b)).

**Figure 2:**
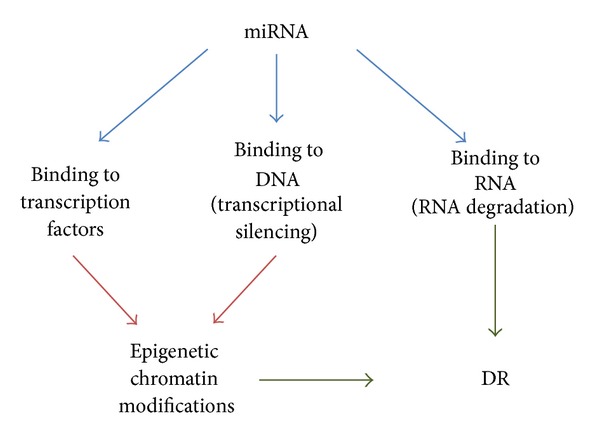
Scheme of miRNA-induced epigenetic modifications. miRNA modulates gene expression in various manners: (i) by directly binding to DNA into the gene promoter region; (ii) by miRNA binding to the 3′-untranslated region of target genes, leading to either posttranscriptional silencing and translational repression or RNA degradation, and miRNAs which can interact with transcription factors and RNA-binding proteins that regulate gene transcription and RNA processing. The binding of miRNA to transcription factors and to DNA regions leads to epigenetic modification of the chromatin, which in turn participate to DR progression.

About ninety percent of the genome is considered to be transcribable though only ~1.5% of the total RNAs represent protein-coding mRNAs [32]. The family of these nontranslated and noncoding RNAs (ncRNAs) includes short interference RNA (siRNA), microRNA (miRNA), Piwi-interacting RNA (piRNA), long noncoding RNA (lncRNA), long noncoding intergenic RNA (lincRNA), and circular RNA (circRNA) [3, 4, 33, 34]. These ncRNAs are critically involved in epigenetics and transcriptome maintenance under various physiological and pathological conditions [32, 35] (Figure 1(a)), especially in terminally differentiated cells such as neurons and retinal pigment epithelium that cannot easily be renewed [36–38]. Among these ncRNAs characterized thus far, the miRNAs are most widely and extensively studied. miRNA modulates gene expression in various manners: (i) by directly binding to DNA into the gene promoter region; (ii) by miRNA binding to the 3′-untranslated region of target genes, leading to either posttranscriptional silencing and translational repression or RNA degradation, and miRNAs which can interact with transcription factors and RNA-binding proteins that regulate gene transcription and RNA processing [39] (Figure 2).

## 5. Signaling Pathways and Metabolic Memory: Key Role of Oxidative Stress

**Figure 3:**
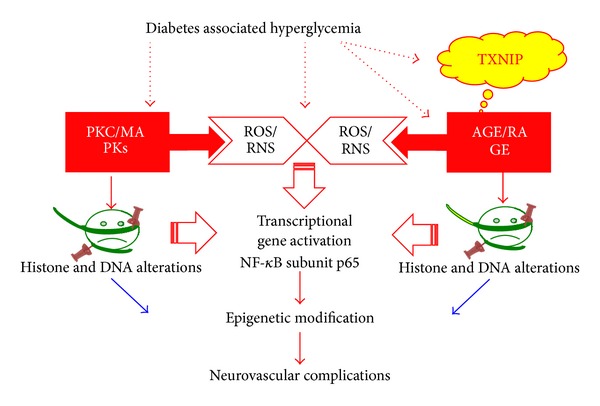
Hyperglycemia-induced pathways leading to epigenetic alteration. Schematic representation of hyperglycemia-induced epigenetic modifications. Hyperglycemia first enhances oxidative damage, which activates several pathways such as the hexosamine pathway, PKC, AGE/RAGE pathway. All these pathways contribute to the modifications of the histone code and activate the NF-*κ*B transcription factor. Moreover, they are inducing alterations in DNA methylation. These modifications lead to epigenetic alteration, which contribute to maintaining the metabolic memory, leading to neurovascular dysfunction in DR.

Common to other diabetic complications, hyperglycemia is also a major risk factor for the development of DR. Hyperglycemia activates several molecular and biochemical pathways that lead to cellular stresses, such as enhanced oxidative/nitrosative stress [16, 40–43], enhanced flux into the polyol and hexosamine pathways [17, 44], activation of PKC [45] and transforming growth factor (TGF) pathway [46], and increased formation of advanced glycation end products (AGEs) and activation of AGEs receptor (RAGE) [47] (Figure 3). Notably, specific blockade of several of these pathways ameliorates DR; however, it is still unclear how these pathways are interconnected and how they may participate in the metabolic memory [48].

Various studies investigated the molecular mechanisms responsible for the metabolic memory in DR. Glycemic control does not block DR in dog models [2]. In agreement, islet transplantation after a prolonged hyperglycemia does not inhibit the progression of DR in rats [2].

Several results support the hypothesis that hyperglycemia-induced oxidative stress plays a central role in promoting the metabolic memory. Indeed, all the signaling pathways mentioned above are activated after hyperglycemia-induced overproduction of superoxide by the mitochondrial electron-transport chain [48]. Oxidative stress and reactive oxygen species (ROS) formation participate in the metabolic memory by activating various pathways (Figure 3).

First, transient hyperglycemia-induced ROS formation in endothelial cells induces epigenetic modifications in the proximal promoter of the NF-*κ*B subunit p65 (Figure 3), leading to the recruitment of Set 7 and the monomethylation of the histone H3 at lysine 4 (H3 K4). These alterations result in sustained increases in p65 gene expression and in the expression of p65-dependent proinflammatory genes, which are long-lasting in the subsequent normoglycemic status [49]. These data underline the dramatic and long-lasting effects induced by a short-term hyperglycemia-induced ROS formation, which may be implicated in the progression of diabetic complications. Hyperglycemia-dependent ROS production leads also to demethylation of histone H3 lysine 9 residue (H3 K9) in the proximal p65 promoter, which reduces the inhibition of p65 transcription and further participating in enhancing p65 gene expression [50].

**Figure 4:**
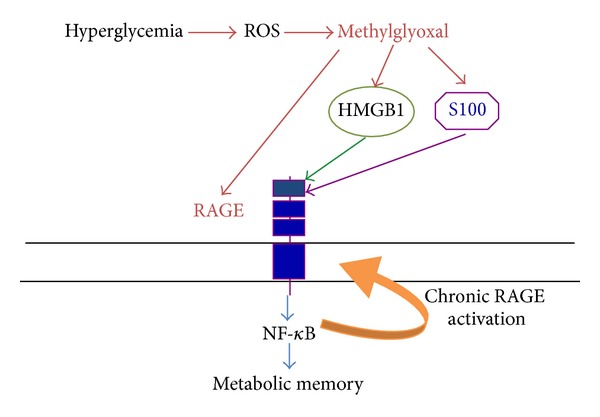
RAGE-dependent metabolic memory. RAGE also contributes to the establishment of the metabolic memory. Hyperglycemia-induced ROS formation leads to the production of methylglyoxal, which in turn induces the expression of RAGE and its ligands S100 and HMGB1. The production of RAGE ligands further activates RAGE, leading to NF-*κ*B activation. NF-*κ*B in turn induces (i) a chronic RAGE activation, leading to chronic neurovascular inflammation; (ii) epigenetic modification of gene expression. Both pathways are implicated in the metabolic memory.

Oxidant stress exerts an important role in perpetuating the metabolic memory also by modifying essential lipids, proteins, and/or DNA [51, 52]. Hyperglycemia and oxidant stress along with increased activity in the polyol pathway and downstream signaling can also increase the accumulation of AGEs, which can further amplify local inflammation and oxidant stress through irreversible glycation of the various proteins and lipids to promote long-term vascular damage [8] (Figure 3). Moreover, AGEs, acting through receptors such as RAGE, could also contribute to hyperglycemic memory, [47, 53, 54]. Recent studies underline the role of the AGEs precursor methylglyoxal, which is formed nonenzymatically from glyceraldehydes 3-phosphate during hyperglycemia [55]. Hyperglycemia-induced oxidative stress leads to methylglyoxal formation, which is sufficient to induce the expression of RAGE as well as its activating ligands S100 calgranulins and HMGB1, leading to a vicious circle of chronic activation of RAGE, which perpetuates the metabolic memory [56] (Figure 4).

Recent studies including our own demonstrated that hyperglycemia induces the expression of thioredoxin interacting protein (TXNIP) [16, 17, 42, 43, 57], which in turn promotes RAGE expression and chronic inflammation [16, 58, 59]. TXNIP is the endogenous inhibitor of the ROS scavenger thioredoxin (Trx) and contributes to hyperglycemia-induced oxidative stress [57, 60, 61]. Notably, TXNIP participates in perpetuating the metabolic memory by inducing epigenetic modifications of gene expression [16], suggesting that oxidative stress may induce epigenetic alterations via TXNIP. Finally, hyperglycemia-induced oxidative stress leads also to mitochondrial DNA damage, which contributes to establishing the metabolic memory [1, 62].

## 6. Epigenetic Alterations in Diabetic Retinopathy

Recent evidences confirm the role for epigenetics in the pathogenesis of diabetic complications [2]. In retinal endothelial cells, a heightened glucose level increases the expression of TXNIP, which promotes epigenetic alterations on the promoter of cyclooxygenase (Cox) 2, inducing Cox2 expression. Silencing of TXNIP completely abrogates epigenetic alterations of the histone code in the Cox2 promoter and its enhanced expression [16]. In addition, hyperglycemia leads to the binding of the histone acetyltransferase p300 to the promoter of TXNIP in the retina of diabetic animals [17]. TXNIP-induced epigenetic modifications play a key role in DR, since inhibition of TXNIP expression blocks DR progression [17]. In this study streptozotocin-diabetic rats were maintained in poor glucose control. Even in the presence of hyperglycemia, inhibition of TXNIP expression prevents both epigenetic alterations and DR progression [16, 17], suggesting that TXNIP may play a key role in establishment and maintenance of the metabolic memory. This study also suggests that TXNIP-induced epigenetic alterations may be implicated in other diabetic complications. Further studies are necessary to investigate this issue. Alterations in the expression of HDAC are implicated in the progression of diabetic complications; thus they participate also in DR [2]. Indeed, in streptozotocin- (STZ-) treated rats, the retinas and retinal endothelial cells (RECs) from animals kept in poor glycemic control (PC) for 6 months show enhanced expression of HDAC1, HDAC2, and HDAC8 and a decrease in the activity of a histone H3-specific acetyltransferase; these changes were not reversed when the PC rats were shifted to good glycemic control for 6 months [63]. These data suggest that the epigenetic alterations in the histone code may be the major reason for the progression of DR even when the blood glucose level returns to normal.

Epigenetic alterations are implicated in the progression of various diabetic complications. However, several studies dissected in detail the effect of epigenetic modifications in affecting specific pathways implicated specifically in DR. In particular, the effect of epigenetic modifications in affecting the role of the manganese superoxide dismutase (product of the sod2 gene) in DR has been analyzed in detail. The sod2 plays a protective role to prevent hyperglycemia-induced damage of mitochondria DNA (mtDNA) in DR [64]. A poor glycemic control of 2 months increases the trimethylation of histone H4 at lysine 20 (H4K20me3) and the acetylation of histone H3 at lysine 9 (H3K9acetyl) and NF-*κ*B p65 at the promoter and enhancer of retinal sod2 and increased the interactions of acetyl H3 K9 and NF-*κ*B p65 to H4K20me3, leading to a decrement in sod2 expression. In rats maintained for 2 months in poor glycemic control followed by 2 months of good glycemic control, reversal of hyperglycemia fails to prevent increases in H4K20me3, acetyl H3 K9, and NF-*κ*B p65 at sod2. Thus, sod2 expression is not restored [65], demonstrating that epigenetic alterations occur after a short time of poor glycemic control. Another study confirmed the relevance of epigenetic changes in sod2 in promoting DR progression by investigating the role of lysine-specific demethylase-1 (LSD1) in sod2 expression* in vivo* in the retina of streptozotocin-diabetic rats maintained for 3 months in poor glycemic control [66]. Hyperglycemia reduces monomethyl histone H3 at lysine 4 (H3K4me1) and dimethyl H3 K4 (H3K4me2), while it increases the binding of LSD1 to sod2, leading to a decrement of sod2 expression [66]. Silencing of LSD1 ameliorates the epigenetic alterations due to hyperglycemia and prevents the downregulation of sod2 expression [66]. In addition, in these rats maintained for 3 months in poor glycemic control, good glycemic control for 3 months failed to revert the epigenetic modifications [66]. These data further confirm the role of epigenetic modifications in the histone code in promoting the metabolic memory and the progression of DR. Detailed studies demonstrated that epigenetic modifications leading to enhanced expression of the matrix metalloproteinase 9 also contribute to mtDNA damage specifically in DR, further promoting the progression of DR [67]. In agreement, a genetic variation in a gene coding for a histone methyltransferase (SUV39H2) is protective for diabetic microvascular complications [68].

In addition, epigenetic alterations in mtDNA are also implicated in the metabolic memory and the chronic progression of DR. In STZ-diabetic rats 6 months of poor glycemic control leads to hypermethylation of the CpG islands in the regulatory regions of the gene encoding the polymerase gamma (POLG1), the catalytic subunit of the mitochondrial DNA replication enzyme. These alterations produce a decrement in the expression of POLG1 [69]. Thus, these studies dissected in detail the effect of epigenetic alterations specifically in DR.

As mentioned before, alterations in miRNA expression induce epigenetic alterations in various diabetic complications. Detailed studies described the effect of specific miRNA in diabetic eyes. We summarize below the role of miRNA specifically in DR progression. For example, when miRNA expression in the retina from rats after 10 weeks of STZ-induced diabetes was compared to untreated rats, changes in expression of 37 miRNAs were detected [70]. Among them, duration differential expression of six of these miRNAs was also confirmed in the retina of STZ-induced diabetic rats. It has been also shown that vascular endothelial growth factor- (VEGF-) induced miR-17–5p, miR-18a, miR-20a, miR-21, miR-31, and miR-133 expression was observed in the retinal endothelial cells (RECs) of STZ-treated rats [71]. The p53-responsive miR-34c was also detected, implicating miRNAs in mediating the proangiogenic or proapoptotic effects caused by VEGF and p53. Reduced miR-200b and increased VEGF have been observed in bovine RECs treated with high glucose. Further, knocking down miR-200b inhibits the diabetes-induced upregulation of p300 in the retina, implying crosstalk between two epigenetic mechanisms in DR [72]. Furthermore, it has been demonstrated that miRNAs may exert a protective role in the early phases of DR. Indeed, miR-29b and its potential target PKR associated protein X (RAX), an activator of the proapoptotic RNA-dependent protein kinase (PKR) signaling pathway, are localized in the retinal ganglion cells (RGCs) and the cells of the inner nuclear layer (INL) of the retinas from normal and diabetic rats [73]. RAX protein is upregulated (more than twofold) at 3, 6, 16, and 22 days after STZ injection and downregulated (70%) at 35 days, whereas miR-29b is upregulated (more than threefold) at 28 and 35 days after STZ injection.

A more recent study also revealed that 11 miRNAs were significantly upregulated and 6 miRNAs were notably downregulated in STZ-induced DR. Levels of miR-182, miR-96, miR-183, miR-211, miR-204, and miR-124 were increased during the progress of DR, whereas miR-10b, miR-10a, miR-219-2-3p, miR-144, miR-338, and miR-199a-3p were decreased [70]. These data further suggest that alterations in miRNA may be implicated in DR progression.

Altered expression of miRNA also has been detected in the retina of the Akita mice, a genetic model of type 1 diabetes. The miR-200b was upregulated significantly in the Akita mouse retina [74]. The target of miR-200b is the oxidation resistance 1 (Oxr1), suggesting that miR-200b has a protective role in DR.

## 7. Role of Epigenetic Alterations in the Metabolic Memory

Several evidences demonstrate the role of epigenetic in the metabolic memory. First, the insulin promoter is methylated in mouse embryonic stem cells, inhibiting the expression of insulin. The insulin promoter is specifically demethylated only in pancreatic *β*-cells, allowing the expression of insulin [2]. Chronic hyperglycemia of diabetes leads to vascular alterations that strongly impact quality of life. DR may be the most common of these and is a leading cause of visual impairment and blindness among working age adults in developed nations. Many large-scale type 1 and type 2 diabetes clinical trials demonstrated that early intensive glycemic control reduces the incidence and progression of micro- and macrovascular complications. On the other hand, epidemiological and prospective data have revealed that the stressors of diabetic vasculature persist beyond the point when glycemic control has been achieved. These kinds of chronic pathologic effects of hyperglycemia on the progression of complications have been defined as “metabolic memory,” and oxidative stress, AGEs, and epigenetic changes have been implicated in the process. Recent studies have indicated that such “hyperglycemic memory” may also influence DR, suggesting that manipulation of hyperglycemic memory may provide a beneficial approach to prevention and treatment. Herein, we summarized the evidence demonstrating the significance of metabolic memory in DR and understand its potential as a target of molecular therapeutics aimed at blocking the hyperglycemic memory. We summarized several evidences demonstrating that epigenetic alterations are responsible for the metabolic memory in DR, since they induce oxidative stress, which is the major player in producing the “metabolic memory.” Indeed, epigenetic modifications participate in the mitochondria damage and are postulated in the development of diabetic retinopathy and also in the metabolic memory phenomenon [62]. These evidences clearly demonstrate the role of oxidative stress in promoting the “metabolic memory,” resulting in DR progression.

## 8. Potential Novel Therapeutic Strategies

Considering the central role of oxidative stress in promoting the metabolic memory in DR by activating several pathways, an antioxidant therapy has been proposed. Indeed, the antioxidant AREDS showed excellent results in diabetic rodents [75]. However, it is still unclear whether a delayed treatment with antioxidant may be capable to revert the epigenetic alterations that occur in DR. In this case, a therapy aimed to revert specifically the epigenetic modifications may be more efficient. It has been shown that DR progression is characterized by enhanced histone acetylation, resulting in modification of the histone code and altered gene expression [76] (Figure 3). Garcinol, an inhibitor of the histone acetyltransferases, blocks these epigenetic modifications as well as DR progression [76], suggesting that this compound can prevent the epigenetic alterations that are implicated in the metabolic memory in DR.

**Figure 5:**
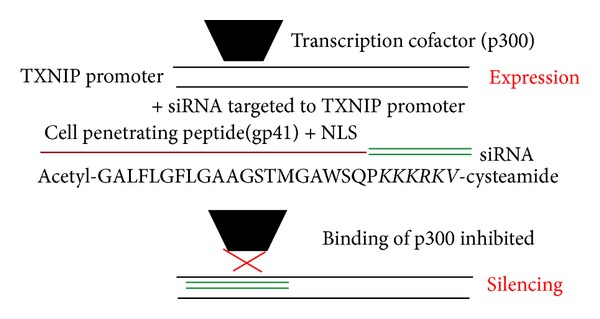
TXNIP: a novel therapeutic target and a novel therapeutic strategy. Hyperglycemia induces TXNIP expression, which leads to oxidative damage that is the major downstream effect of hyperglycemia. Indeed, we demonstrated that TXNIP is responsible for neurovascular dysfunction in DR. We developed a novel gene therapy technique: we injected in the diabetic retina small interfering RNA (siRNA) targeted to TXNIP promoter associated with cell penetrating peptides (CPP) containing a nuclear localization signal. In diabetic conditions, the cotranscriptional factor p300 is associated with TXNIP promoter, leading to enhanced TXNIP expression. When we injected the siRNA targeted to TXNIP promoter, they completely block the recruitment of p300 on TXNIP promoter, inhibiting TXNIP expression. With this technique, we completely blocked the progression of DR.

However, a more gene-targeted therapy could yield a better specific effect in inhibiting DR progression. A novel gene therapy technique that efficiently inhibits the early pathologies in DR has been established: the epigenetic silencing of TXNIP in the diabetic retina [17]. TXNIP is early induced by hyperglycemia* in vitro* and diabetes* in vivo* diabetic retina. TXNIP plays a central role in promoting neurovascular dysfunction in DR and the epigenetic silencing of TXNIP prevents DR progression [16, 17] (Figure 4). This technique consists in injecting in the retina of STZ diabetic rats small interfering RNA (siRNA) targeted to the TXNIP promoter and coupled* via *electrostatic bounds (not covalent) with cell penetrating peptides (CPP) containing a nuclear localization signal, in order to target the delivery of the siRNA into the cell nucleus and the TXNIP gene regulatory region [17]. These siRNAs completely block the recruitment of the acetyltransferase p300 on the TXNIP promoter region, resulting in a total inhibition of TXNIP expression in the diabetic retina even in hyperglycemic conditions [17] (Figure 5). TXNIP silencing* in vivo* using this technique completely prevents early molecular abnormalities of DR, which include retinal inflammation, capillary basement membrane thickening, gliosis, and ganglion cell death [17]. These data suggest that TXNIP is a therapeutic target for DR. Moreover, this novel gene therapy method for epigenetic gene silencing does not present the possible side effect of an* in vivo *gene-silencing therapy using retrovirus. Both the siRNA and CPPs are short-lived compounds and they do not incorporate into host genome. The use of this technique targeting disease-associated genes in a stage-specific and duration-dependent manner may be very important when poor glycemic control occurs and effects of the metabolic memory are established. Indeed, for a delayed treatment, a more gene-targeted or miRNA targeted therapy may be necessary. Considering the recent data showing that miRNA plays a role in DR progression, this technique may be used to restore the expression of specific miRNA in late DR, by coupling these miRNA with CPP.

## 9. Conclusion

We briefly summarized in the review article recent studies that contributed to the elucidation of potential molecular mechanisms responsible for the metabolic memory in DR progression. Although chronic hyperglycemia-associated oxidative stress plays a central role in promoting aberrant molecular and biochemical signaling pathways and cellular damage that are implicated in DR, recent evidences also suggest that metabolic defects resulting in altered generation of epigenetic substrates, such as acetyl-CoA and S-adenosylmethionine, will also change epigenetic chromatin modifications (Figures 1 and 3). Such epigenetic alterations may play a critical role in DR pathogenesis, thereby, opening the way for the discovery of novel therapeutic strategies. Finally, the emerging role of miRNA and other ncRNAs suggest that novel gene-targeted small inhibitory RNA strategies including siRNAs (Figure 5) and miRNAs may be employed. Furthermore, the role of long noncoding nuclear RNAs in chromatin reprogramming under chronic nutrient excess and DR will be critically important. Equally, drugs targeting histone deacetylases and methylases will need further exploration in preventing or slowing down the progression of DR.
